# Long acting growth hormone (LAGH), an update

**DOI:** 10.3389/fped.2023.1254231

**Published:** 2023-09-28

**Authors:** Margaret Steiner Grillo, Jacklyn Frank, Paul Saenger

**Affiliations:** Pediatric Endocrinology, NYU Langone Hospital-Long Island, Mineola, NY, United States

**Keywords:** growth hormone, long acting growth hormone, pegylation, fusion proteins, growth hormone deficiency

## Abstract

In 1957, Maurice Raben at Yale was able to isolate and purify growth hormone from cadaveric pituitary glands. Pituitary growth hormone was the only way to treat children with growth hormone (GH) deficiency, until 1985 when recombinant GH became available for daily subcutaneous injection. For many years, the pediatric endocrine community longed for a long-acting recombinant GH formulation that would decrease the inconvenience of daily injections. Several mechanisms were employed to develop a GH that is rapidly absorbed into the blood stream after subcutaneous injection, but provides slow removal from the circulatory system to potentially optimize patient adherence to GH therapy. Four long-acting growth hormones are currently available in the world, or are close to regulatory approval. They are: (1) Pegylated formulations, (2) Prodrug formulations which are converted into active drug, (3) Nonvalent transient albumin binding GH compounds and (4) GH fusion proteins where a protein si fused with GH. All four formulations have undergone detailed phase 3 studies and were found to show non-inferiority in these clinical studies. All four demonstrate a safety and tolerability profile that is comparable to that of daily somatropin with an excellent adherence profile.

## Introduction

In the late 1950s, Raben (1, 2) was able to isolate and subsequently purify growth hormone (GH) from cadaveric human pituitaries. However, its clinical use was restricted due to the limited availability of supplies, and the main beneficiaries were a limited number of children with GH deficiency (GHD). For the next 40 years, all GH was provided in the U.S. by the National Pituitary Agency free of charge. In 1985, the first patients with lethal Creutzfeldt-Jakob disease led to an immediate stop in the use of pituitary-derived GH (3). Fortunately, recombinant DNA-generated GH became available in that same year. Recombinant DNA technology became the standard to produce a large supply of native, 22-kDa, 191 amino-acid long human GH (hGH), and hundreds of thousands of patients benefited from the growth-promoting and metabolic effects of hGH on the human body (2, 4, 5).

There is a complex regulatory system that controls the pulsatile release of GH bursts into the peripheral circulation approximately every 3 h (6–8 discrete pulses daily). Many pulses occur during slow-wave sleep. The majority of smaller pulses occur during times other than slow-wave sleep (stage 3 sleep). Even though GH-releasing hormone and somatostatin have been recognized as the main modulators of this axis, there are a number of other key players that seem to be important to achieve the optimal biological effects of this hormone including ghrelin, glucocorticoids, thyroid hormones, nutritional and pubertal status, bone age, as well as other metabolic and age-related mechanisms (2, 6, 7). This complex physiological regulation system has not only a theoretical interest, but also clinical implications. The clinical practice across the world (three times per week, or daily) documents that none of the previously used or currently recommended GH regimens to treat GHD in pediatric or adult populations are actually physiological. Daily GH is not physiological, and long acting growth hormone is therefore also not physiological. As evidence shows, both work to improve height. Nevertheless, these non-physiological replacement therapies have many years of safety data records and have documented that they promote linear growth in children without major safety concerns and excellent metabolic effects in children and adults (2).

Daily subcutaneous injections are at least as effective to generate insulin-like growth factor 1 (IGF-1) production and promote linear growth as earlier attempts to treat GHD patients with twice or thrice weekly intramuscular injections, continuous intravenous infusions, or even more “physiological” pulsatile replacement (8, 9). The current standard daily regimen also implies adherence concerns, particularly in the long-term, multi-year use of daily GH (2, 8, 9), and adherence particularly may falter after years of GH therapy, leading possibly to suboptimal auxological outcomes (10).

GH replacement therapy is used to treat GHD (10), and until recently, most treatments required once-daily injections. This routine can be burdensome for children and their parents/guardians, affecting adherence and leading to suboptimal clinical outcomes (2, 4, 11). Factors contributing to poor adherence include the burden of daily injections, pain caused by injections, storage issues, the need to reconstitute solutions, and managing injections while traveling (6, 12, 13). There is, therefore, a need for GH therapies that require a reduced injection frequency, while still being effective and having a tolerable safety profile compared to daily hGH (14).

Three key issues will be addressed in this brief review.

1. What is the evidence that height velocity SDS will be maintained and even possibly improved when switching from daily to LAGH.
2. What is the evidence that treatment burden is indeed reduced when GH is administered weekly instead of daily.
3. To date, all published studies demonstrate non inferiority of LAGH to daily GH.

Weekly administration of GH reduces the number of GH injections from 365 to 52 injections. Thus, it is expected to reduce treatment burden, minimize disruption of patients' lives, and potentially improves treatment adherence (15). To date, evaluations and data of treatment burden have been reported for two LAGH formulations, namely Somapacitan and Somatrogon (11, 13).

In two studies (NovoNordisk and Pfizer) (11, 13) questionnaires were developed and validated to assess the burden that GHD and GH treatment can have on patients and their parents/guardians. The GHD-child-impact-measure (CIM) questionnaire can be used to assess disease burden in children with GHD (5, 7, 8). The GHD-Child-Treatment-Burden (CTBD) and GHD-Parent-Treatment-Burden (PTB) questionnaires can be used to assess treatment burden in patients and their parents/guardians, respectively (11, 13). In a 2nd study, life input questionnaire was developed and 81.8% strongly or very strongly preferred the LAGH to daily.

Switching from daily to long acting growth hormone is well tolerated with no attenuation in height velocity (14). Data from this review show a dramatically reduced treatment burden. The quest for long acting medication regimens has been on the forefront of medical innovation for quite some time. For instance, central precocious puberty is treated with long acting GnRH agonists that have a duration of action from 3 months to even 1 year. Multiple other drug therapies, including insulins, Cabergolide, PTH, HIV antivirals, and certain antipsychotics take advantage of an extended duration of action to increase convenient use and adherence.

### Overview of long acting GH preparations

Patients, parents, and providers have yearned for long acting drugs for decades. The list is long and growing. Long acting drugs families include insulins, Cabergolide, PTH, antiviral medications, psychiatric medications, long acting GnRH agonists, long acting metformin and many others (17) (Table 1).

**Table 1:** Summary of LAGH product Development history (17).

Company	Product	Modification to GH molecule (Molecular weight)	Frequency of administration	Current status	Research
PEGylated formulations		PEGylation prolongs *in vivo* mean residence time of GH, though slowing absorption and protection from proteolysis.			
GeneScience Pharmaceuticals Co, Ltd	Jintrolong	40-kDa PEG attached to GH	7 days	Marketed in China for CGHD	Phase 3 studies show good IGF-1 profile
Prodrug Formulation	Mechanism of conversion to active drug				
Ascendis	TransCon GH (Skytrofa)	Unmodified rhGH transiently bound to a PEG carrier molecule via a self-cleaving linker that is dependent upon pH and temperature (22 kDa)	7 days	Phase 2 studies in CGHD and AGHD showed comparable IGF-1 profile to daily GH dosing Phase 3 studies in CGHD show positive growth response and was approved for treatment of children with GH in the fall of 2021 in the US.	Phase 3 study in CGHD ongoing and phase 3 study in AGHD planned. SGA and Turner studies are planned
Noncovalent albumin binding GH compound(s)		Albumin binding			
Novo Nordisk A/S	Somapacitan (NNC0195-0092)	Single-point mutation in GH, with albumin binding moiety attached (noncovalent albumin—binding properties) (23 kDa)	7 days	Phase 2 studies in CGHD showed comparable IGF-1 profile to daily GH dosing Phase 3 studies in AGHD well tolerated	Phase 3 studies in CGHD and extension study in AGHD ongoing
GH fusion proteins		Protein fused with GH			
OPKP Health and Pizfer	Somatrogon (MOD-4023)	rhGH fused to 3 copies of carboxy-terminal peptide of hCG B-subunit (47.5 kDa)	7 days	Phase 2 studies in CGHD Phase 3 studies in AGHD did not meet primary endpoint	Approved in Europe and Canada

AGHD, adult growth hormone deficiency; CGHD childhood growth hormone deficiency; rhGH recombinant human GH.

Steiner M, Frank J, Saenger P. Long-acting growth hormone in 2022. Pediatr. Investig 2023;00,1–7 (17).

Permission has been obtained to duplicate.

For many years, the pediatric endocrinology community has longed for long-acting recombinant hGH (rhGH) formations that would decrease the inconvenience of daily injections and potentially optimize patient's compliance with such therapy. Over the last two decades, this has now finally became a reality.

A LAGH should, at minimum, must have the same excellent efficacy and safety profile as GH administered daily while also reducing the number of injections (15). All LAGH preparations should aim for a once-weekly treatment for GHD. It reduces the injection frequency from 365 injections per year required for daily GH replacement to 52 injections per year (9, 18).

### History of LAGH development

Lippe et al. (2, 19) studied the utilization of intramuscular GH gel (15%) as a depot hGH formation twice per week with similar growth results compared to a thrice-weekly regimen of the standard aqueous GH solution during the 1st year, but a waning effect of growth velocity was noted in the 2nd year of treatment, even after adjusting dosing by weight.

Genentech developed in 1999 a LAGH preparation Nutropin Depot, which was approved for the treatment of GHD. The preparation was unmodified GH linked to biodegradable microspheres which led to a sustained GH release over 4 weeks. The children showed catch-up growth and IGF-1 peaked at 14–17 days. There were adverse reactions such as atrophy and nodules at the injection sites, a very painful injection, large injection volumes, and over 1 ml in children above 30 kg body weight necessitated multiple injections. Achieved growth velocities were not as robust as daily GH. Manufacturing issues plagued the product and the product was discontinued in 2004 (20). Nutropin Depot was only approved in the U.S.

A prototype LAGH was developed by LG Life Sciences and very successful height data were published in 2014 (2, 21, 22). This prototype was approved by the European Medicines Agency for Europe in 2016, however, has not been marketed except in South Korea.

Multiple formulations of LAGH are currently at advanced stages of development. Three have been recently approved by regulatory agencies in the U.S. and elsewhere (Lonapegsomatropin (Ascendis), Somatrogon (Pfizer), and Somapacitan (NovoNordisk)) in randomized non-inferiority trials (12, 13, 23).

The benefit of LAGH is to decrease the burden of injections, as GH currently is given daily. The hope for LAGH is to decrease the frequency from daily to weekly. LAGH preparations include molecular changes to the weight and ionic charge, as well as binding to other molecules. These molecular changes could affect the way the molecule interacts with the intended target tissues. Furthermore, the peak GH and IGF-1 levels may vary based on the formulation. Some LAGH preparations have undergone randomized clinical control studies and are non-inferior in terms of height velocity and body composition to the daily rhGH injections.

The use of LAGH in place of daily rhGH will be feasible now, however, there are still questions that need to be answered. Dose adjustments, the timing of IGF-1 serum level monitoring, safety, and long-term evaluation of metabolic parameters of LAGH action. Efficacy, and cost-effectiveness all need to be further evaluated. LAGH will need long-term post-marketing surveillance before it is safely used as a replacement for daily rhGH injections.

Mechanisms that have been explored for LAGH action include formulations that create a subcutaneous depot which allows for native or modified GH to slowly diffuse into surrounding tissues and vasculature. The other mechanisms explored include preparations that are rapidly absorbed into the bloodstream but provide slow removal from the circulatory system (17) (Table 1).

### Jintrolong

Jintrolong is an irreversibly PEGylated LAGH and has been approved in China (Gene Sciences). Clinical trials have been performed in children using weekly Jintrolong, and it has been found to produce high levels of GH. Phase 3 trials in children have shown higher IGF-1 levels in comparison to daily rhGH, and a good height velocity (24, 25). Currently, Jintrolong is being used extensively in China to treat childhood GHD. Reported efficacy and safety are identical to daily rhGH (24, 25). It should be noted that all long-acting PEGylated GH preparations were abandoned in Europe and the U.S. and the European Medicines Agency published a critical review of PEGylated GH (26).

### Lonapegsomatropin a reversibly PEGylated pro-dug

**Figure 1:**
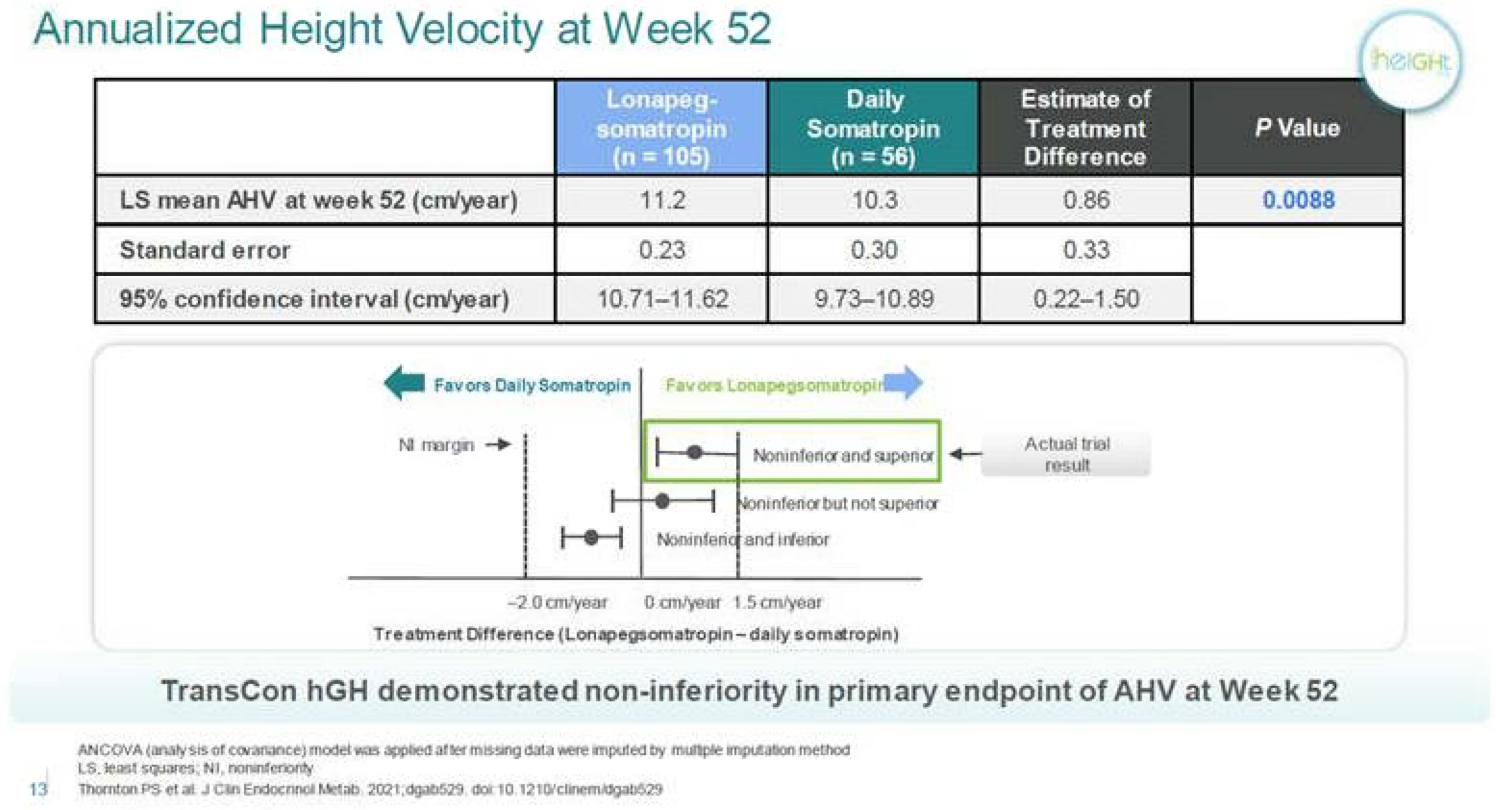
Annualized height velocity of lonapegsomatropin compared to daily Somatropin. Personal communication from Ascendis Pharma, and reference (13).

**Figure 2:**
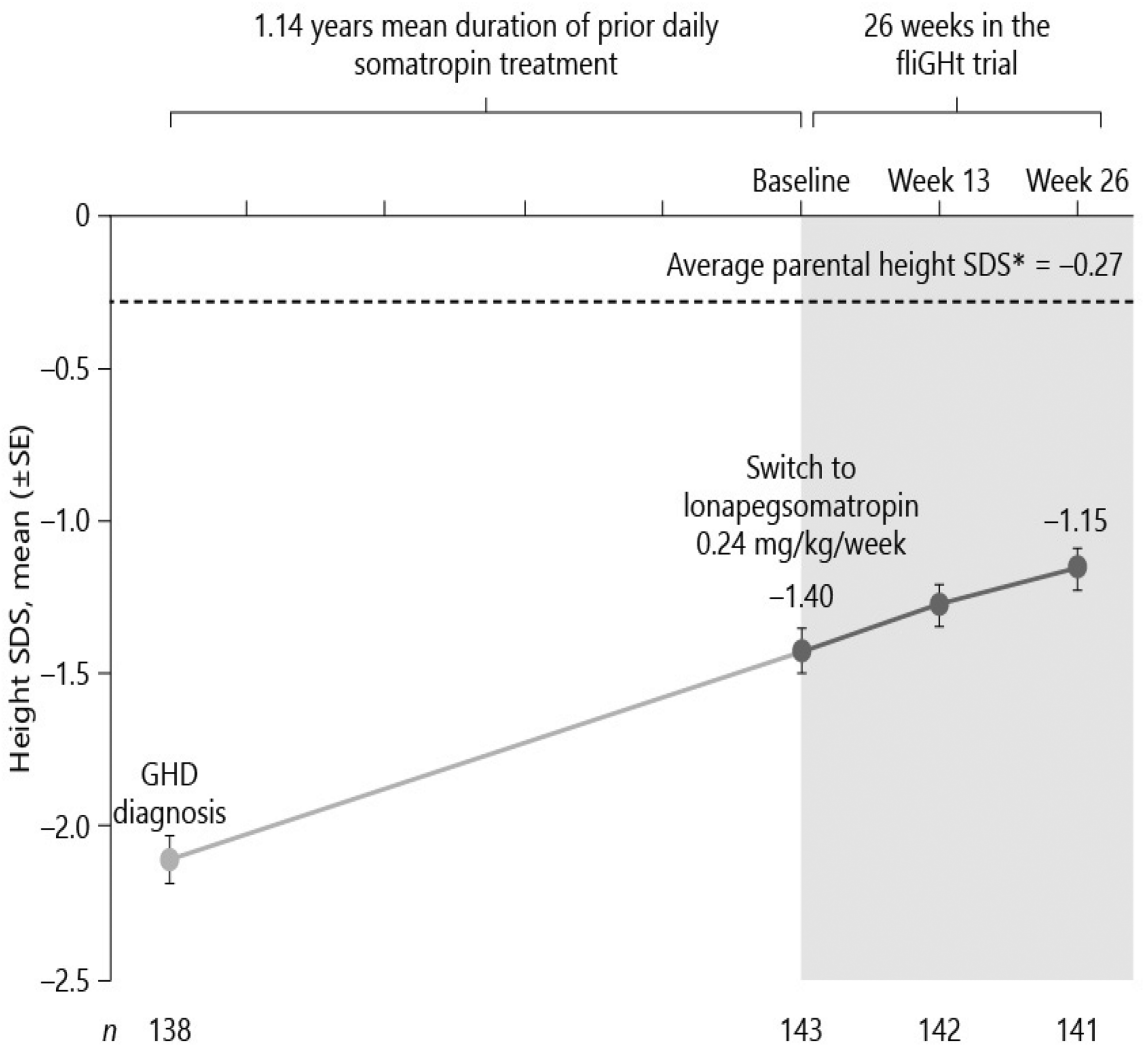
Switching to weekly lonapegsomatropin from daily somatropin in children with growth hormone deficiency (15). Permission has been obtained to duplicate.

Lonapegsomatropin (Ascendis) (13) is a reversible, transiently PEGylated GH. This reversibility leads to the release of an unmodified rhGH. Lonapegsomatropin has completed phase 3 clinical trials, and it has been shown to have a superior height velocity in comparison to daily rhGH injections. There have been so far no identified safety concerns, and no antidrug antibodies have been reported (13) (Figures 1, 2).

Lonapegsomatropin-tcgd (Skytrofa) is a once-weekly treatment for GHD. The Food and Drug Administration (FDA) approval of lonapegsomatropin was based on an open-label trial in 161 treatment-naïve prepubertal children with GHD, defined as a peak serum GH ≤ 10 ng/ml (13). Patients were required to have a height standard deviation score (SDS) of ≤2.0. Annualized height velocity at 52 weeks, the primary endpoint, was 11.2 cm/year in the lonapegsomatropin group and 10.3 cm/year in the somatropin group. Lonapegsomatropin met the prespecified criteria for noninferiority and superiority compared to somatropin. Change in height SDS from baseline, a secondary endpoint, increased by 1.1 with lonapegsomatropin and 0.96 with somatropin (13, and Table 1).

A clinical trial evaluating lonapegsomatropin for adults with GHD is ongoing, no GH antibodies, and more importantly, no neutralizing antibodies were found in clinical studies (13).

The FDA has approved, in the fall of 2021, lonapegsomatropin-tcgd (Ascendis), LAGH for once-weekly treatment of growth failure due to inadequate secretion of endogenous GH in children ≥1 year old who weigh ≥11.5 kg. It is the first once-weekly treatment approved for CGHD since Nutropin Depot.

Lonapegsomatropin is therefore a long-acting prodrug of somatropin that consists of somatropin bound to an inert methoxypolyethylene glycol (mPEG) carrier by a proprietary transient conjugation (TransCon) linker. The mPEG carrier minimizes renal excretion and receptor-mediated clearance of the drug. Under physiologic conditions, the methoxypolyethylene glycol carrier is cleared by the kidneys, the linker is hydrolyzed, and therapeutic levels of somatropin are released over one week. The half-life of the drug is approximately 25 h compared to about 3 h for somatropin. Lonapegsomatropin is designed to release somatropin with the identical 191 amino acid sequence and size (22 kDa) as both endogenous GH and daily somatropin therapy; thus, the released somatropin is expected to maintain the same mode of action, distribution, and intracellular signaling (13, 27).

### Somapacitan

**Figure 3:**
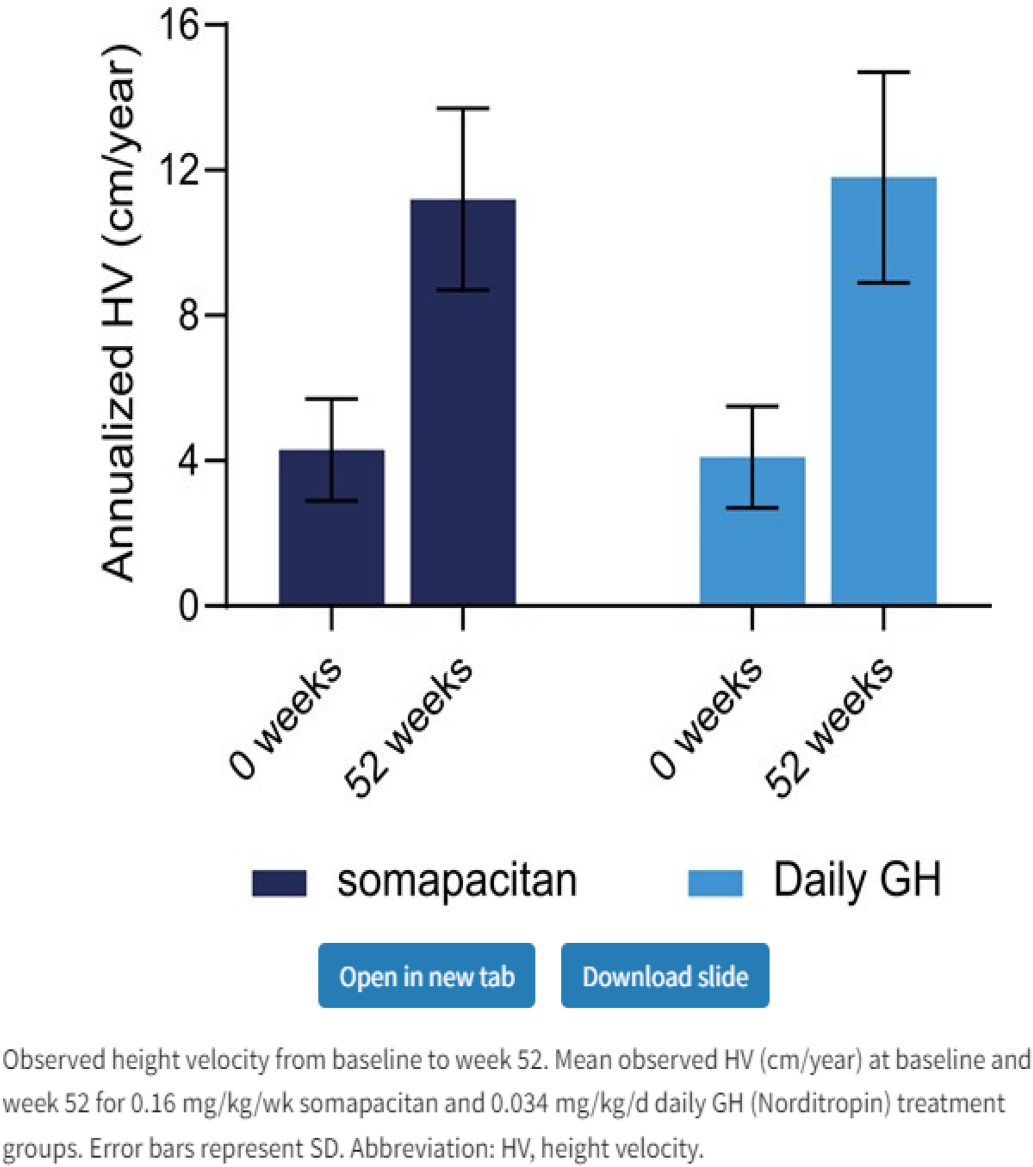
Weekly somapacitan is effective and well tolerated in children with GH deficiency (19). Permission has been obtained to duplicate.

Somapacitan (Novo Nordisk) is a modified GH. The addition of one amino acid imparts a higher affinity to bind to endogenous albumin. Somapacitan is a 23.2-kDa human GH derivative (99% similarity to endogenous GH) linked to a small noncovalent albumin-binding moiety that facilitates reversible endogenous albumin binding to delay somapacitan elimination. Similar technologies to enhance the half-life of other peptide drugs, such as long-acting insulin detemir, glucagon-like peptide-1 molecules liraglutide (28, 29), and semaglutide (30). In previous trials, somapacitan has been shown to be well tolerated in adults and children with GHD (19, 30–34) and effective in adults with GHD. A phase 2 dose-finding and safety trial in prepubertal children with GHD suggests 0.16 mg/kg/week somapacitan has the same efficacy and safety profile as daily GH treatment (0.034 mg/kg/d Norditropin, Novo Nordisk A/S) for up to 3 years of treatment (19, 32–34). Somapacitan has also been shown to decrease fat composition in comparison to daily GH when used in adults (34). During phase 2 and phase 3 studies in CGHD, somapacitan and daily GH had similar IGF-1 values, and it was found that somapacitan had an improved height velocity in comparison to daily rhGH. Savendahl et al (33–35). recently report a height velocity SDS for somapacitan of 8.6 compared to daily GH therapy of 7.4 (Figures 2, 3).

Somapacitan has recently been approved for adult GHD and pediatric use. In adult and pediatric GHD, no antibodies to GH or neutralizing antibodies to GH were found. The molecular weight of somapacitan is 23 kDa, thus very close to native GH, which is 22 kDa (33, 34).

### Fusion proteins

Fusion proteins have been used in the development of rhGH structure to prolong the half-life of the molecule as well as to decrease the clearance of rhGH from circulation. The lingering concern with fusion proteins is that it increased the molecular weight of the molecule, which could potentially hinder the absorption of rhGH into target tissues. GH structure and size are highly conserved among various species from fish to man with molecular weights ranging from 19.4 to 22 kDa. Studies with labeled dextran show a 40 kDa molecular weight cut-off for diffusion into the growth plates of mice (36, 37). Fusion proteins prolong the half-life and reduce the renal clearance of rhGH, but may dramatically increase molecular weight (36, 37), which may affect tissue penetrance.

The conservation of size may represent evolutionary control to allow GH to transit less well-vascularized tissues (fat, bone, growth plates). Theoretically, GH analogs >40 kDa, e.g., VRS-317 (38, 39), an abandoned LAGH studied by Versartis, may be capable of generating hepatic IGF-1, but not able to activate lipolysis in adipose tissues or promote the entry of resting chondrocytes into the proliferative zone of the growth plate. Thus, large GH fusion proteins may create a response that is more characteristic of IGF-1 therapy with sub-optimal growth and increased fat mass/body mass index (36–40). However, the ability of LAGH to reach different target tissues may also depend on characteristics other than molecular size, including the charge of the molecule (35–38). The fact that the VRS-317 (40) product did not meet non-inferiority criteria in clinical trials may have been due to the large molecular size of 115 kDa (40).

Somatrogon (MOD-4023; Pfizer) is a fusion protein with a weight of 47 kDa. It contains the amino acid sequence of hGH and three copies of the C-terminal peptide (CTP) (41) of human chorionic gonadotropin which has a molecular weight of 47 kDa and 275 amino acids. It was previously shown that the inclusion of CTP proteins, such as follicular stimulating hormone (41–43) and erythropoietin (41–43) led to increased drug half-life. Height velocity was 10 cm/year compared to 9.8 cm/year for subjects treated with daily GH (23, 44).

Somatrogon is a long acting GH compromising the amino acid sequence of hGH fused to three copies of the carboxy-terminal peptide from human chorionic gonadotropin (hCG) (41–43). The carboxy-terminal peptides from hCG extend the half-life of the attached hGH (41) allowing longer intervals between injected doses. Somatrogon has 84 additional amino acids resulting in a total of 275 amino acids. The molecular weight ranges between 34 and 77 kD, depending on the amount of glycosylation in the molecule, making the molecule considerably larger.

Somatrogon is currently approved in Canada, Australia Japan, the UK and the EU but not yet in the US (see letter from OPKO health) (45).

## Considerations when planning LAGH therapy

As LAGH use becomes more prevalent, there are issues to still consider. As of now, it is unknown what the long-term metabolic consequences and side effects are of LAGH. The molecular composition of LAGH and rhGH differ, and it is unknown if there will be differences in metabolism between the two molecules. Currently, it is recommended to monitor routine IGF-1 values while on rhGH therapy with e.g., lonapegsomatropin on day 4.5 after the injection (46, 47), however there is no such recommendation for monitoring other biomedical markers of therapy, such as carbohydrate metabolism in patients on LAGH.

Safety profile is similar to that of daily injection for the time period of the clinical trials with the longest follow-up reaching 5 years. Furthermore, post-marketing studies for all LAGH products approved and marketed will provide much-needed real-world data (14).

It is also important to consider the cost-benefit analysis of rhGH and LAGH therapies. As of now, it is not yet known if LAGH is a more cost-effective treatment in comparison to rhGH. As the first US-approved LAGH from Ascendis is coming to the market, the price of above $120,000 for one year of treatment for an average child is considerably higher than daily GH and should be reduced. Price for the other two LAGH are not available at this point.

Although a weekly injection may provide ease of administration when compared to daily injections, we do not yet know if LAGH will indeed improve compliance with GH therapy compared to daily injections of rhGH. Preliminary data by the Brod group and others (48, 49) are interesting as they show that treatment burden was significantly diminished (14). There are no data detailing if and how LAGH increased compliance in comparison to daily GH. These studies are in progress.

It should be noted that all studies used daily GH as comparators and used a daily GH dose of 0.025 mg/kg/day. This is less than the widely used US dose of 0.042 mg/kg/day. The European Medicines Agency set these daily GH doses for all LAGH studies performed to date.

Changes from baseline height velocity and height velocity SDS were similar and as expected in both groups. Observer-reported outcomes showed that patients and parents/guardians seem to have experienced a reduced treatment burden when switching from daily GH to somapacitan. Most parents/guardians (81.8%) strongly/very strongly preferred somapacitan over daily GH (48, 49). Many patients will switch from daily GH to LAGH. This successful switch was addressed in two studies (Ascendis and Novo Nordisk).

### Metabolic effects of LAGH

GH affects body composition via opposing GH (lipolytic) and IGF-1 (adipogenic) effects. Additionally, GH increases relative lean body mass by decreasing protein oxidation and increasing protein synthesis in skeletal muscle. A key concern in the development of LAGHs has been that modified GH formulations may result in variable tissue distribution due to molecular weight (50). Non-physiological tissue distribution can result in increased serum IGF-1 levels due to GH activity in the liver but a lack of GH effects in size-restricted target tissues such as fat. Therefore, elevated IGF-1 levels in adipose tissue in the absence of GH's lipolytic effects may result in an adipogenic effect, which can result in net fat accumulation and weight gain.

Somatropin released from the lonapegsomatropin prodrug is unmodified and is expected to exhibit a pattern of tissue distribution and affinity for the GH receptor identical to that of endogenous GH and daily somatropin therapies. Indeed, treatment with lonapegsomatropin in this long-term extension was associated with mean BMI SDS that stabilized toward 0 (50).

There was no increase in bone age advancement with lonapegsomatropin therapy, indicating that the longer-term effects of lonapegsomatropin (up to 104 weeks) did not occur at the expense of accelerated skeletal maturation. It follows then that improvements in near-final height could be anticipated with lonapegsomatropin treatment (12).

Values for glucose, hemoglobin A1c, and insulin remained in the normal range. Effects on visceral fat need to be evaluated (23, 50).

### Immunogenicity

Analyses of immunogenicity are ongoing. Antidrug antibodies are frequent, up to 77% (44), they had no effect on safety or efficacy. No antidrug antibodies had so far shown evidence of neutralizing activity which could have an effect on safety or efficacy. Analyses of immunogenicity are ongoing as part of the open label extensions of these studies (44).

### Treatment burden

The two treatment burden analyses document a lower treatment burden for Somatrogon, which was approved in the US by the FDA on June 28,2023 (Ngenla), when measuring life impact scores (13, 16, 48, 49, 51). Prior studies have shown that poor treatment adherence is associated with a suboptimal treatment response as well as economic cost (13). Similar data accrued from a study reporting on 4 years Somapacitan treatment/disease burden observer reported outcomes showed that patients and parents seem to have experienced a reduced treatment burden when switched from daily GH to LAGH. Most parents, namely 81.8% strongly/very strongly preferred Somapacitan over daily GH.

## Outlook

The major benefit of LAGH is to decrease the burden of injections, as GH currently is given daily. The hope for LAGH is to decrease the frequency from daily to weekly and then to monthly. LAGH preparations include molecular changes to the weight and ionic charge, as well as the binding to other molecules. These molecular changes could affect the way the molecule interacts with the intended target tissues. Furthermore, the peak GH and IGF-1 levels may vary based on the formulation. There have not to date been additional adverse reactions noted from LAGH compared to rhGH.

The evidence to date suggests that all LAGH preparations covered in this review will achieve similar growth rates to one year growth rates in treatment naïve pre-prepubertal children on daily growth hormone. All trials were conceptualized and carried out as non-inferiority trials. Ascends reported a slightly higher HV in a 1 year trial with treatment naive trial or after switching from daily rhGH to weekly GH. LAGH resulted in slightly higher IGF-1 levels of approximately 0.3 SD (Figure 1). Slightly higher IGF-1 levels were seen in all reported LAGH preparations.

The use of LAGH in place of rhGH could be feasible in the future, however there are still questions that need to be answered. Dose adjustments, the timing of IGF-1 serum level monitoring (14, 46), safety, efficacy, insurance approval, and cost-effectiveness all need to be further evaluated. LAGH will need long-term surveillance before it is used as a replacement for daily rhGH injections. In particular, IGF-1 monitoring is important in all LAGH preparations. Recent research by Lin et al. (46) provides a formula to predict IGF-1 levels. They recommend IGF-1 sampling 4.5 days after dosing. This goal may be difficult to achieve in the clinical setting, therefore, phase 4 studies evaluating IGF-1 levels in children on LAGH are mandatory.

To date, the only US-approved LAGH was not associated with increased adverse events, immunogenicity, or metabolic complications. Only a low incidence of GH-binding antibodies but no neutralizing antibodies were observed following lonapegsomatropin treatment.

Switching to weekly LAGH from daily rhGH is well tolerated and maintains the known high safety profile of daily rhGH (14), as shown in a recently published study.

The clinical use of GH is an exciting success story beginning with pituitary-derived GH in the mid-1950s to the regulatory approval of recombinant DNA-generated GH in 1985. We now have as of 2022 several new drugs in development for LAGH for children and adults with GHD. Children achieve similar height outcomes, or in some studies, even better one-year height data compared to daily use in children. This is the real success story.

Much-needed improved adherence and safety data will further solidify the use of LAGH in clinical medicine, as we predicted in a 2016 consensus paper (18).

## Summary

Recent publications (12, 19, 44) support robust 5-year efficacy and safety results previously published for all 4 LAGH in the treatment of prepubertal children with GHD. Outcomes and safety profiles are needed for naive patients and for those switched from daily GH to LAGH. Observer reported outcomes suggest LAGH may pose a reduced treatment burden for patients and parents. Nonetheless, phase 4 post marketing studies will hopefully become an integral part of continued drug surveillance. Lessons learned from daily GH approved in 1985 described side effects of GH reported after initial approval of daily GH when very large cohorts of patients were treated in long term post marketing studies for longer than 10 years.
